# The influence of aortoseptal angulation on provocable left ventricular outflow tract obstruction in hypertrophic cardiomyopathy

**DOI:** 10.1136/openhrt-2014-000176

**Published:** 2014-10-30

**Authors:** Christopher Howell Critoph, Antonios Pantazis, Maria Teresa Tome Esteban, Joel Salazar-Mendiguchía, Efstathios D Pagourelias, James C Moon, Perry Mark Elliott

**Affiliations:** 1 Department of Inherited Cardiovascular Disease, The Heart Hospital, University College London, London, UK; 2 Cardiomyopathies, Advanced Heart Failure and Transplant Unit, Hospital Universitari de Bellvitge, Barcelona, Spain

## Abstract

**Objectives:**

Aortoseptal angulation (AoSA) can predict provocable left ventricular outflow tract obstruction (LVOTO) in patients with symptomatic hypertrophic cardiomyopathy (HCM). Lack of a standardised measurement technique in HCM without the need for complex three-dimensional (3D) imaging limits its usefulness in routine clinical practice. This study aimed to validate a simple measurement of AoSA using 2D echocardiography and cardiac MR (CMR) imaging as a predictor of LVOTO.

**Methods:**

We retrospectively assessed 160 patients with non-obstructive HCM, referred for exercise stress echocardiography. AoSA was measured using resting 2D echocardiography in all patients, and CMR in 29. Twenty-five controls with normal echocardiograms were used for comparison.

**Results:**

Patients with HCM had a reduced AoSA compared with controls (113°±12 vs 126°±6), p<0.0001. Sixty (38%) patients had provocable LVOTO, with smaller angles than non-obstructive patients (108°±12 vs 116°±12, p<0.0001). AoSA, degree of mitral valvular regurgitation and incomplete systolic anterior motion (SAM) were associated with peak left ventricular outflow tract gradient (r=0.508, p<0.0001). An angle ≤100° had 27% sensitivity, 91% specificity and 59% positive predictive value for predicting provocable LVOTO. When combined with SAM, specificity was 99% and positive predictive value 88%. Intraclass correlation coefficient of AoSA measured by two observers was 0.901 (p<0.0001). Bland-Altman analysis of echocardiographic AoSA showed good agreement with the CMR-derived angle.

**Conclusions:**

Measurement of AoSA using echocardiography in HCM is easy, reproducible and comparable to CMR. Patients with provocable LVOTO have reduced angles compared with non-obstructive patients. AoSA is highly specific for provocable LVOTO and should prompt further evaluation in symptomatic patients without resting obstruction.

**Key Messages**

**What is already known about this subject?**

- Transthoracic echocardiography can be used to measure aortoseptal angulation. However, data using this technique in patients with hypertrophic cardiomyopathy and its associated geometric abnormalities are lacking. It has been suggested, using three-dimensional (3D) imaging techniques, that aortoseptal angulation is an important determinant of left ventricular outflow tract obstruction.

**What does this study add?**

- This study modified the echocardiographic technique, and validates a standardised method of aortoseptal angulation measurement that can be used in patients with hypertrophic cardiomyopathy without recourse to complex 3D imaging. It also demonstrated that a reduced aortoseptal angle is highly specific for provocable left ventricular outflow tract obstruction and should prompt further evaluation in symptomatic patients without resting gradients.

**How might this impact on clinical practice?**

- We have demonstrated that our methodology for aortoseptal angle quantification using standard 2D transthoracic echocardiography provides a simple, quick, relatively inexpensive, robust method, which provides additional information that may be of clinical benefit to patients with hypertrophic cardiomyopathy. We propose that reduced aortoseptal angle be considered to serve as a ‘red flag’ for the presence of provocable left ventricular outflow tract obstruction and prompt further specialist stress imaging.

## Introduction

Hypertrophic cardiomyopathy (HCM) is the commonest inherited cardiac disease with a population prevalence of 1 in 500.^1^ Left ventricular outflow tract obstruction (LVOTO) caused by systolic anterior motion (SAM) of the mitral valve leaflets is present in one-third of patients at rest,^2^ and occurs in up to two-thirds of symptomatic patients without resting obstruction during manoeuvres that reduce preload and afterload or increase contractility.^3^ The mechanism of SAM varies between patients, but in most individuals it represents a complex interaction between altered left ventricular (LV) chamber shape, mitral leaflet length and orientation of the mitral and submitral apparatus.^4–6^ Recently, it has been suggested that aortoseptal angulation is an important determinant of LVOTO that can be used as a predictor of provocable obstruction in symptomatic patients without resting left ventricular outflow tract (LVOT) gradients.^7^ However, the lack of a standardised method of measurement that can be used in patients with HCM without recourse to complex three-dimensional (3D) imaging techniques limits the usefulness of this parameter in routine clinical practice. Transthoracic echocardiography is widely available, and can be used to measure aortoseptal angulation. However, data using this technique in patients with HCM and its associated geometric abnormalities are lacking. The aims of this study were to validate a simple measurement of aortoseptal angulation using 2D echocardiography and cardiac MR (CMR) imaging and to determine its relation to provocable LVOTO in patients with HCM.

## Methods

### Patient cohort

This was a retrospective cohort study. The study population comprised consecutive patients with non-obstructive HCM referred for exercise stress echocardiography between August 2004 and December 2008. All patients fulfilled conventional diagnostic criteria for HCM and had symptoms or signs consistent with provocable obstruction. None of the patients had received interventional gradient reduction therapy (myectomy or alcohol ablation). Contemporaneous peak oxygen consumption measured during symptom limited upright bicycle ergometer exercise testing, functional class and medication were recorded in all patients. The control group consisted of 25 age-matched and sex-matched individuals referred for transthoracic echocardiography to investigate symptoms of chest pain or breathlessness, who were subsequently found to have normal studies, with no history of hypertension, myocardial or valvular heart disease.

### Transthoracic echocardiography

Resting transthoracic echocardiography was performed using vivid i7 (General Electric Vingmed Ultrasound, Horten, Norway) and Philips Sonos 7500 (Philips Medical Systems, Andover, Massachusetts, USA) platforms using standard acquisition protocols. Exercise echocardiography was performed using the same equipment during symptom-limited exercise on an upright bicycle ergometer using a ramp protocol. Echocardiographic parameters were measured according to European Society of Echocardiography guidelines,^8–10^ using EchoPAC (General Electric) software. Basal interventricular septal thickness was measured in the parasternal short-axis view. SAM was defined as incomplete if there was any movement of the mitral valve leaflets or chordae towards the ventricular septal endocardium without septal contact and complete when there was contact with the ventricular septum during systole. Mitral regurgitation was graded visually as none, mild, moderate or severe at rest and during provocation.^9^ Maximal LVOT gradient was measured using continuous wave Doppler in the apical five-chamber view at rest, during and immediately after exercise. Care was taken to exclude Doppler signals from mitral regurgitation and mid-cavity obstruction. Resting LVOTO was defined as a peak LVOT gradient ≥30 mm Hg, and provocable LVOTO was defined as a gradient ≥50 mm Hg during or immediately following exercise. The smallest LVOT diameter below the aortic valve annulus during ventricular systole was measured in the parasternal long-axis view.

### Measurement of aortoseptal angle

**Figure 1:**
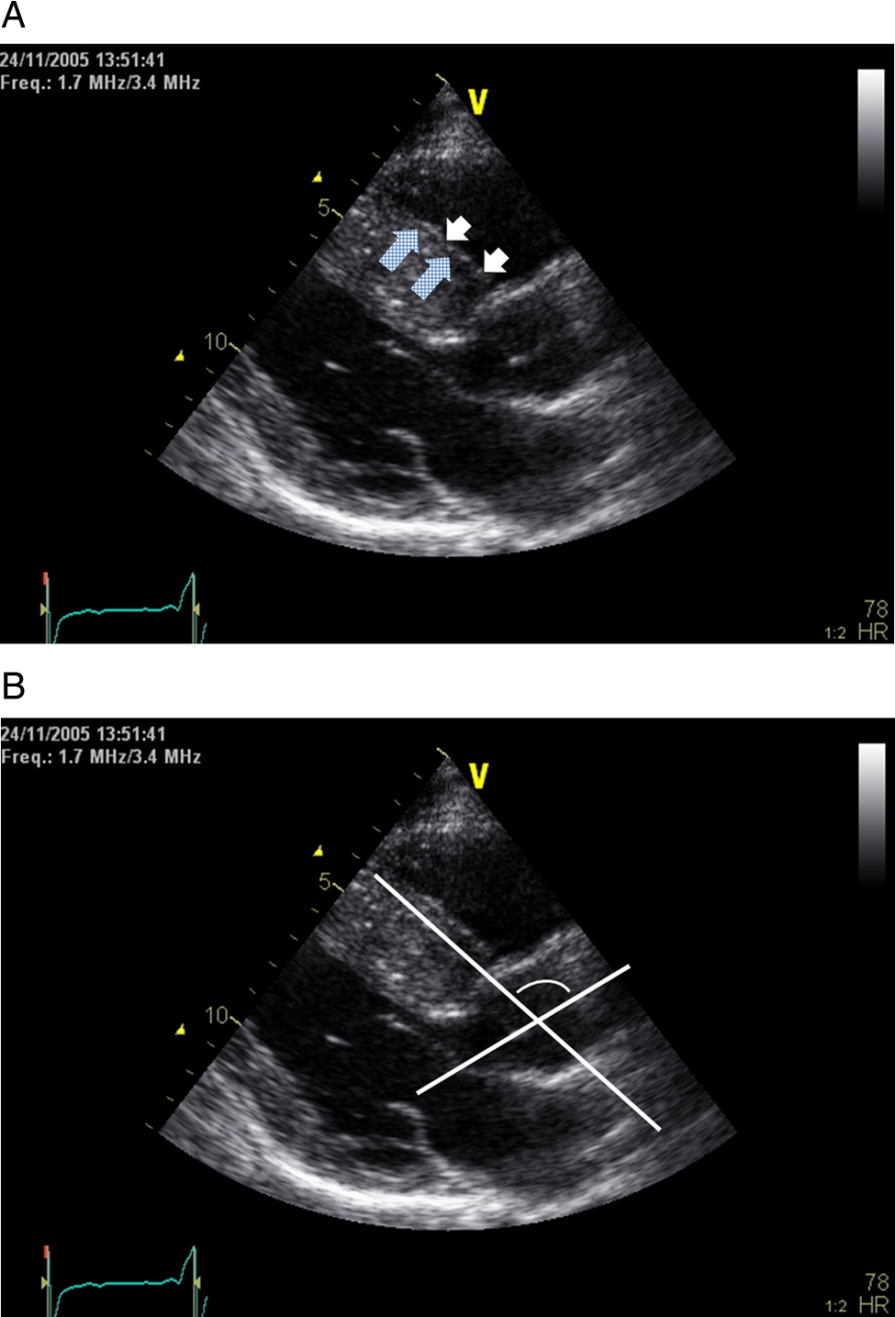
Transthoracic echocardiogram, parasternal long-axis view: example of construction of reference lines for aortoseptal angle calculation. (A) The septal line was drawn along the junction of left and right interventricular septum (checked arrows), parallel to the proximal right endocardial border (white arrows). (B) The aortoseptal angle was defined as the angle between the septal line, and a line drawn through the long axis of the aortic root where a value of 180° would be a straight line from septum to aorta and reducing values represent increasing angulation.

A parasternal long-axis view taken at the R wave of the surface ECG was used for analysis. The image was analysed offline using a DICOM image viewer (SOBOX V.2.3.0.1). The aortoseptal angle was measured using a modification of the technique originally described by Fowles *et al*,^11^ and defined as the angle between a line drawn along the border of the right and left interventricular septum (parallel to the proximal right ventricular endocardial border), and a line drawn through the long axis of the aortic root (figure 1), where a value of 180° would be a straight line from septum to aorta and reducing values represent increasing angulation.

Two cardiologists trained in echocardiography and cardiomyopathy independently evaluated all images. In 29 patients, contemporaneous CMR images were available, and 3D data sets were loaded onto a standard offline work station (Leonardo, Siemens Medical Solutions) for analysis of the LV-aortic root angle. This angle was measured in a multiplanar reformatted LVOT view (intended to replicate the echocardiographic images) using the same reference lines as for echocardiography.

### Statistics

Normally distributed variables are presented as mean±SD, and non-normally distributed data as median and IQR. Analysis was carried out using SPSS statistical software V.19 (SPSS for Windows, IBM, USA). For all tests a p value <0.05 was considered significant. The mean value of the angle measured by two observers was used as an independent variable and assessed alongside the above additional echocardiographic parameters using a linear regression model to determine the univariate associations of the peak provocable LVOT gradient. Significant factors were then entered into a stepwise elimination model to determine multivariate predictors. A similar model was used to determine the associations of the aortoseptal angle. Binary logistic regression and receiver operator characteristic curve analysis was used to determine the sensitivity and specificity of the aortoseptal angle alone or in combination with resting incomplete SAM to detect the presence of provocable LVOTO. Differences between two groups were assessed using independent two-sample t test. One way Analysis of variance (ANOVA) with homogeneity of variance testing, and post-hoc Bonferroni or Games-Howell corrections were used as appropriate to assess differences between multiple groups. Interobserver variability was assessed using a two-way mixed model intraclass correlation coefficient (absolute type) and Pearson's correlation. Agreement between echocardiography and CMR measured angles was assessed using Pearson's correlation coefficient and Bland-Altman analysis.

## Results

### Patient characteristics

The initial study cohort comprised 179 patients. On the day of exercise, 19 individuals were found to have a LVOT gradient ≥30 mm Hg at rest and were excluded from the analysis. The final study cohort therefore consisted of 160 patients (105 males, age 48±14 years). Descriptive demographic and echocardiographic characteristics are shown in table 1. Twelve (8%) patients were unable to perform exercise and underwent measurement of the LVOT gradient following the administration of sublingual glyceryl trinitrate in combination with Valsalva manoeuvre. Fifty-nine (37%) patients were taking a β-blocker or calcium channel antagonist as they were unable to discontinue before the test for symptomatic reasons; a further 40 (25%) patients withheld these drugs for a minimum of 48 h prior to study. There was no difference in aortoseptal angle between those who were either not on or withheld medication and those who took it on the day of exercise (113°±12 vs 114°±12, p=0.709). In patients who were unable to discontinue medication for symptomatic reasons, there was a trend towards being more likely to develop provocable obstruction (p=0.07). Twelve (20%) patients with and 10 (10%) without provocable LVOTO were in New York Heart Association (NYHA) class III (p=0.097). There was no difference in provocable LVOTO or aortoseptal angle (108°±12 vs 114°±12, p=0.12) in those with or without hypertension, although prevalence was low.

**Table 1:** Patient demographics, clinical and echocardiographic characteristics

Demographics and baseline data		
Age (years)	50 (19), range 16–82	
Male gender	105 (66%)	
Height (cm)	173 (14)	
Weight (kg)	82±16	
Peak oxygen consumption (mL/kg/min)	19.0 (11.4)	
Per cent predicted peak oxygen consumption	67 (32)	
Hypertension	14 (9%)	
Medication		
Calcium antagonist or β-blocker on day of test	59 (37%)	
Calcium antagonist or β-blocker withheld >48 h pretest	40 (25%)	
NYHA functional class		
2	138 (86%)	
3	22 (14%)	
Echocardiographic parameters		
Basal septal thickness (mm)	16±4	
LVOT systolic diameter (mm)	19±3	
Aortoseptal angle (degrees)	113±12 (range 79–140)	
Left ventricular end diastolic diameter (mm)	46±6	
Distribution of hypertrophy		
Asymmetric	146 (91%)	
Concentric	10 (6%)	
Apical	4 (3%)	

	**Rest**	**Provocation**

LVOT gradient (mm Hg)		
Whole cohort	7 (6)	28 (69)
<30	160 (100%)	81 (51%)
30–49	–	19 (12%)
50–69	–	12 (8%)
≥70	–	48 (29%)
SAM		
None	82 (51%)	62 (39%)
Incomplete	78 (49%)	39 (24%)
Complete	0	59 (37%)
Mitral regurgitation		
None	19 (12%)	17 (11%)
Mild	139 (87%)	120 (75%)
Moderate	2 (1%)	20 (13%)
Severe	0	3 (2%)

Normally distributed data mean±SD, non-parametric data median (IQR).

LVOT, left ventricular outflow tract; NYHA, New York Heart Association; SAM, systolic anterior motion.

### Aortoseptal angle

Patients with HCM had a smaller aortoseptal angle than controls (113°±12 vs 126°±6, p<0.0001). There was no difference in aortoseptal angle between men and women (112°±13 vs 114°±11, p=0.297). There was a weak negative correlation between aortoseptal angle and age (r=−0.242, p=0.002) and also height (r=−0.181, p=0.036). There was no relationship between aortoseptal angle and body weight. There was no correlation between aortoseptal angle and basal septal thickness or LVOT systolic diameter.

### Relationship between echocardiographic variables and provocable LVOTO

Univariate analysis of echocardiographic variables and their relationship to peak provocable LVOT gradient is shown in table 2. Aortoseptal angle (β −1.18; CI −1.68 to −0.68; p<0.0001), incomplete SAM at rest (β 30.59; CI 18.15 to 43.04; p<0.0001) and degree of resting mitral regurgitation (β 20.61; CI 2.64 to 38.59; p=0.025), overall r=0.508, p<0.0001, were independently associated with peak provocable LVOT gradient on multivariate analysis. There was no difference in left ventricular end-diastolic dimensions between patients with and without provocable LVOTO.

**Table 2:** Univariate predictors of peak provocable LVOT gradient

			CI		
Factor	r	β	Lower	Upper	p Value
Aortoseptal angle	0.319	−1.165	−1.708	−0.622	<0.0001
Basal septal thickness	0.048	−0.613	−2.618	1.392	0.547
Incomplete SAM (rest)	0.366	33.06	19.861	46.259	<0.0001
Mitral regurgitation grade (rest)	0.197	25.604	5.535	45.677	0.013
LVOT systolic diameter	0.014	−0.24	−2.949	2.469	0.861

LVOT, left ventricular outflow tract; SAM, systolic anterior motion.

**Figure 2:**
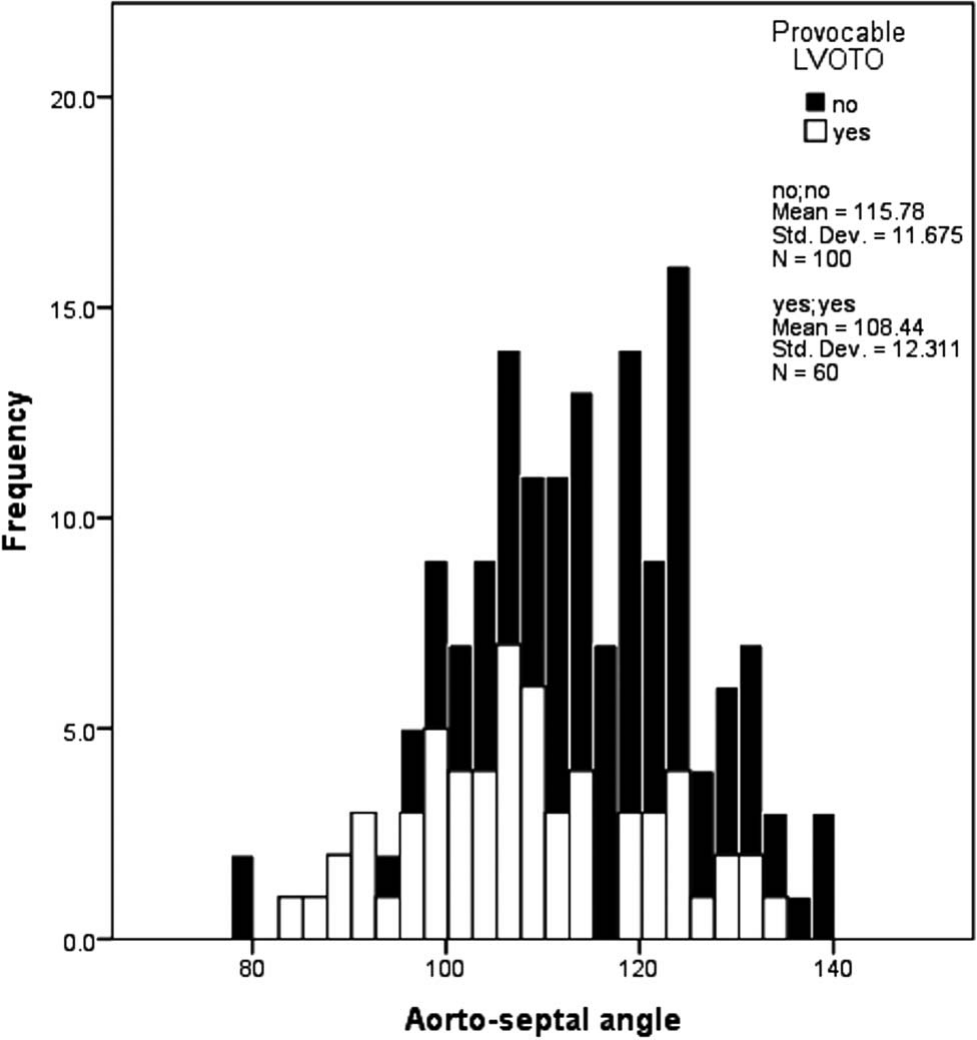
Histogram showing variation in aortoseptal angle between patients with hypertrophic cardiomyopathy with and without provocable left ventricular outflow tract obstruction (LVOTO).

**Figure 3:**
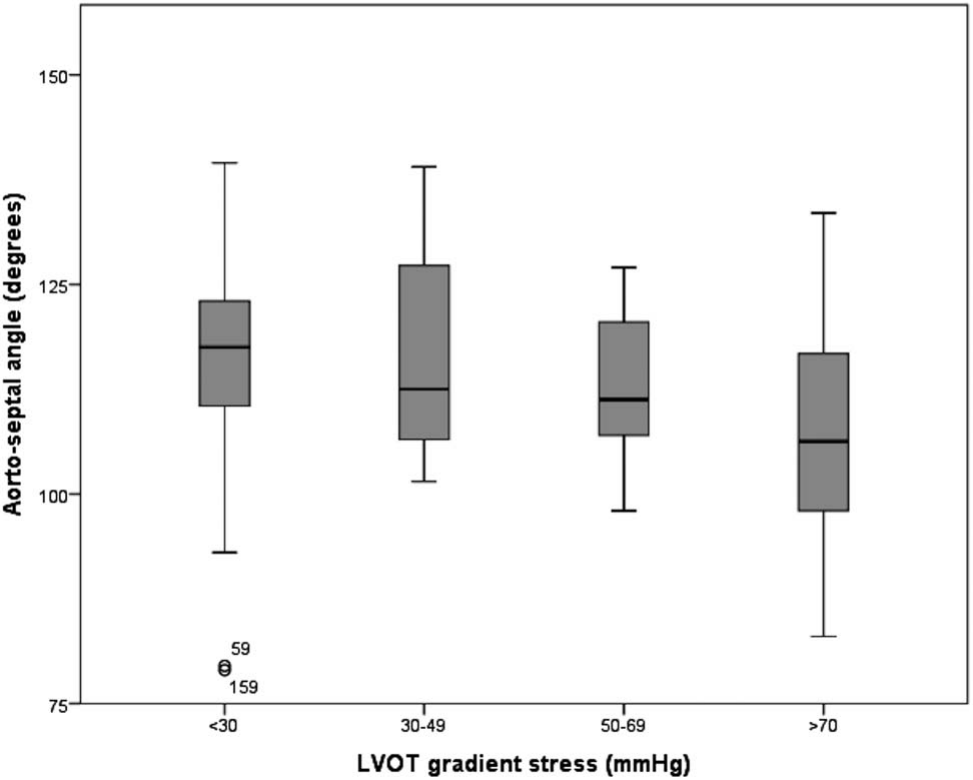
Box plot showing decreasing aortoseptal angle with increasing severity of provocable left ventricular outflow tract (LVOT) gradient.

The aortoseptal angle was smaller in patients with provocable LVOTO (108°±12 vs 116°±12, p<0.0001), figure 2. When grouped by LVOT gradient, a smaller aortoseptal angle was found in patients with increasingly severe LVOTO, p=0.004, figure 3.

**Figure 4:**
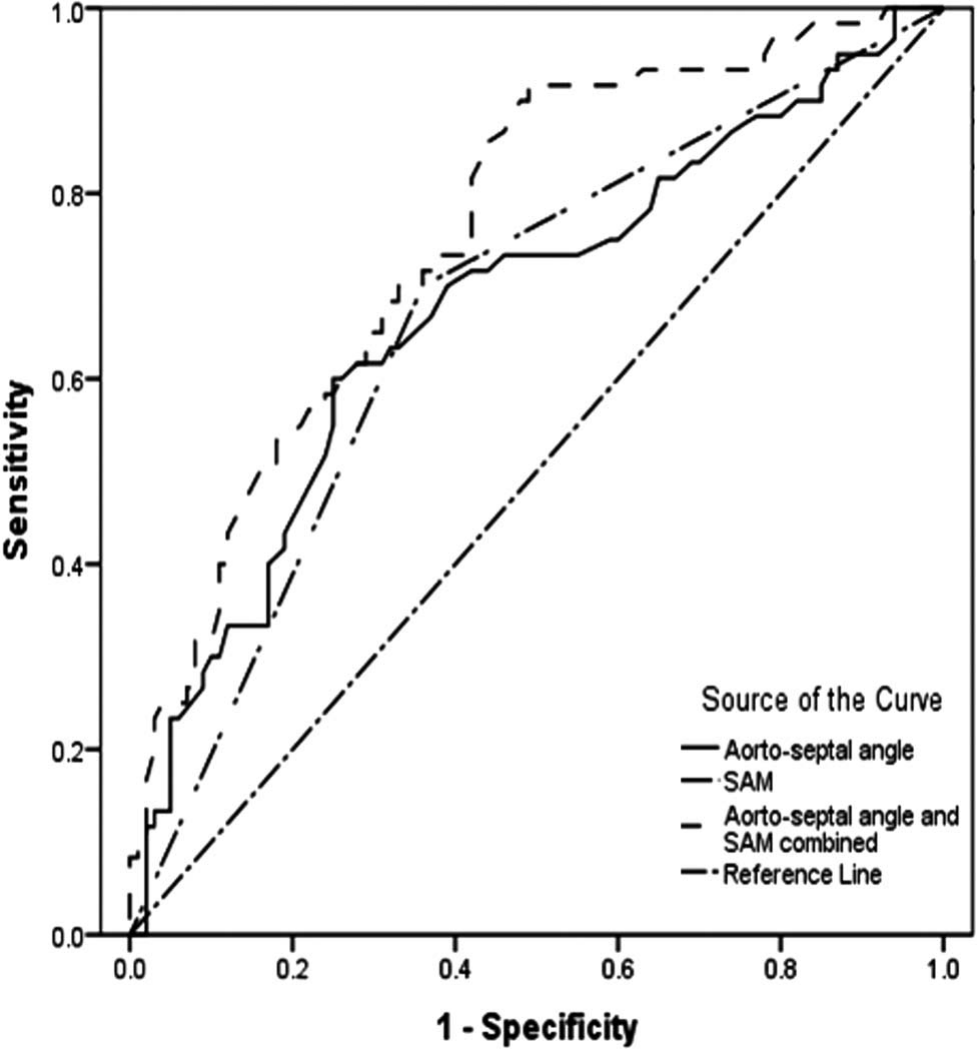
Receiver operator characteristic curves showing the probability that aortoseptal angle, presence of systolic anterior motion (SAM) of the mitral valve and both combined predict patients who develop provocable left ventricular outflow tract obstruction during exercise.

The area under receiver operator curves for aortoseptal angle, incomplete rest SAM, and the two parameters combined for the prediction of provocable LVOTO were 0.68 (95% CI 0.59 to 0.76, p<0.0001), 0.67 (95% CI 0.58 to 0.76, p<0.0001 and 0.76 (95% CI 0.68 to 0.83, p<0.0001), respectively (figure 4). Sensitivity, specificity and positive predictive value for SAM and aortoseptal angulation are shown in table 3.

**Table 3:** Sensitivity, specificity and positive predictive value to predict provocable left ventricular outflow tract obstruction in patients with hypertrophic cardiomyopathy using resting echocardiographic parameters

	Sensitivity (%)	Specificity (%)	Positive predictive value (%)
Incomplete SAM rest	70	64	54
Aortoseptal angle ≤100° N=25 (16%)	27	91	59
Aortoseptal angle ≤100° and incomplete SAM rest N=8 (5%)	12	99	88
Aortoseptal angle ≤110° N=37 (23%)	62	71	56
Aortoseptal angle ≤110° and incomplete SAM rest N=19 (12%)	23	95	74

SAM, systolic anterior motion.

#### Reproducibility

**Figure 5:**
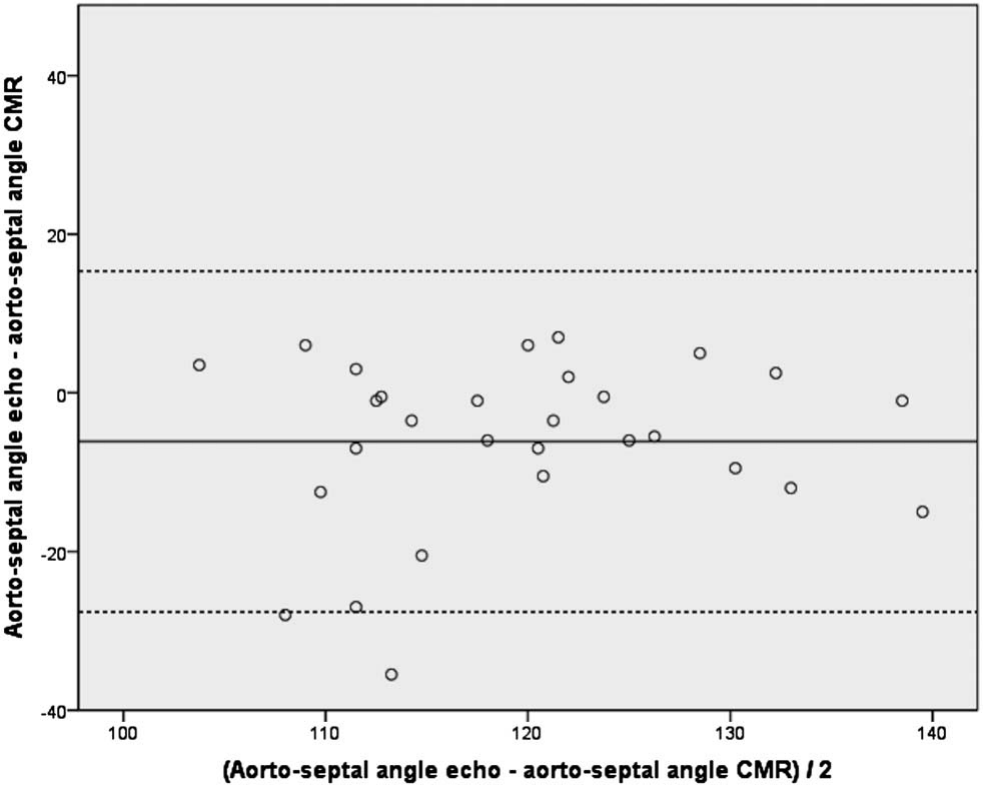
Bland-Altman plot of the differences between aortoseptal angulation measured using transthoracic echocardiography and cardiac MR (CMR) imaging. Solid line represents mean, dashed line represents mean ±2 SDs.

All images were deemed usable for the purpose of angle measurement by both observers. The intraclass correlation coefficient of the aortoseptal angles measured by the two observers was 0.90 (95% CI 0.87 to 0.93, p<0.0001), with Pearson's coefficient r=0.82, p<0.0001. Correlation between the aortoseptal angle measured using echocardiography and CMR was r=0.50, p=0.006. The mean difference between the echocardiography angle−CMR angle was −6° (SD 11). A Bland-Altman plot of the differences in angle measured using the two modalities plotted against their mean is shown in figure 5.

## Discussion

The main findings of this study are that patients with HCM have a smaller aortoseptal angle than controls, and individuals who develop provocable LVOTO during exercise have a smaller angle than those without obstruction. The angle can be easily and reliably measured using standard 2D transthoracic echocardiography in patients with HCM, and is more specific than the presence of SAM for the identification of provocable LVOTO.

### Determinants of provocable LVOTO and aortoseptal angulation

A variety of structural features are associated with LVOTO including anterior displacement of papillary muscles,^12^ reduced LVOT area^13^ and primary mitral valve abnormalities,^14–16^ but the essential component in the majority of patients is contact between the mitral valve and the interventricular septum caused by SAM of the anterior, and, less commonly, the posterior leaflets. Incomplete SAM at rest has long been recognised as a clue to the presence of provocable LVOTO, but this study demonstrates for the first time that reduced aortoseptal angle has a higher specificity when used as a single measure. The relatively low sensitivity of both parameters is expected given the high prevalence and complexity of LVOTO, which is related to many additional factors.

Variation in LVOT geometry is characteristic of HCM, as well as of aortic valve disease and hypertension in which a ‘sigmoid’ configuration is common, particularly with advancing age and reduction in LV cavity size.^17–21^ In adults, a smaller aortoseptal angle is associated with increased aortic pressure wave reflection and higher central blood pressure,^22^ although no causal relationship has been demonstrated to date and in children a smaller aortoseptal angle is a highly sensitive, specific and positive predictive marker for the development of subaortic stenosis,^23^ and can be used as an echocardiographic feature to identify individuals at risk.^24^

Data on the importance of LVOT diameter in predicting patients with provocable obstruction are conflicting possibly due to differences in methodology.^3^ ^25^ ^26^ Owing to the dynamic nature of provocable LVOTO, measurement of this parameter may often be distal to the point of SAM-septal contact and relatively fixed, and therefore may not accurately reflect 3D LVOT area. The lack of an unequivocal association is not therefore surprising. We measured the LVOT diameter using 2D images in the parasternal long-axis view and found no influence on the presence or magnitude of LVOT gradient.

### Technique of aortoseptal angulation measurement

CMR and CT imaging provide a 3D measurement of aortoseptal angulation, which has been shown to predict LVOTO provoked using Valsalva or amyl nitrite independent of basal septal thickness.^7^ A variety of methods have been used for measurement of the aortoseptal angle using 2D transthoracic echocardiography. The most commonly adopted is the angle formed by the long axis of the ascending aorta and the plane of the ventricular septum, which has excellent interobserver correlation.^11^ ^24^ ^27^ ^28^ While 2D echocardiography has been used multiple times to measure aortoseptal angulation, it has not been evaluated in HCM. The left ventricular endocardial border in HCM rarely forms a straight line, particularly in patients with a basal septal bulge. We recognised the difficulties of finding consistent echocardiographic landmarks from which to accurately quantify the aortoseptal angle from standard 2D images in HCM, and so attempted to determine reference lines that would be most consistently applicable across echocardiographic studies and between observers. We therefore modified the method originally described by Fowles *et al*.^11^ Instead of using a line bisecting the septum at the level of the mitral valve leaflets and 2 cm apically, we constructed one at the junction of the left and right interventricular septum, parallel to the proximal right endocardial border. We hypothesised that this technique would be a more accurate representation of the true septal orientation in HCM and more likely to fit a straight line. We believe the major determinant of the angle in HCM to be abnormal septal orientation, with less variability seen in the position of the aorta. The quality of echocardiographic images varies between individuals, and in a minority of patients fit to a straight line can be challenging. However, the results of our interobserver analysis support the reliability of our method across a large number of studies, and comparison with CMR data shows good agreement.

### Clinical relevance and applicability

Patients with refractory symptoms and resting LVOTO should be considered for myectomy,^29^ ^30^ or alcohol septal ablation.^31–33^ Provocable LVOTO is associated with functional impairment and heart failure symptoms,^34–36^ and there is good evidence that invasive treatments should be offered to these patients.^37^ ^38^ Therefore a simple clinical tool that helps identify patients at risk of developing provocable LVOTO would be of benefit.

We propose that reduced aortoseptal angle be considered to serve as a ‘red flag’ for the presence of provocable LVOTO. If suspected, it can be quickly and easily quantified, and even in the absence of resting SAM on 2D echocardiography should prompt further evaluation with stress echocardiography. Availability of expertise for stress echocardiography in patients with HCM is variable, although it can be performed safely,^39^ and as such the relatively low sensitivity of aortoseptal angle measurement demonstrated here is of less importance. However, the high specificity is likely to identify patients who may then benefit from treatment. As used here, current guidelines to diagnose provocable LVOTO recommend use of either a treadmill or bicycle in combination with Doppler echocardiography during and/or immediately following exercise, as simple manoeuvres such as Valsalva may underestimate the gradient.^30^

We have demonstrated that our methodology for aortoseptal angle quantification using standard 2D transthoracic echocardiography provides a simple, quick, relatively inexpensive, robust method that is comparable to MR and provides additional information that may be of clinical benefit to patients with HCM. Furthermore, the results may be equally relevant in other patients with reduced aortoseptal angle, for example, those with hypertension, and the elderly. Further study in these groups is warranted.

## Limitations

This is a retrospective observational study on a consecutive cohort of patients selected for stress echocardiography from a specialist cardiomyopathy clinic. We did not assess specific abnormalities of the mitral valve, anterior displacement of the papillary muscles or LVOT area. Only symptomatic patients were referred for stress echocardiography, and so the relevance of aortoseptal angulation in asymptomatic patients is unknown. One-third of patients were unable to discontinue medication for symptomatic reasons; this reflects real-world practice, but may have underestimated the prevalence and magnitude of LVOTO.

Basal septal thickness was relatively modest in our cohort. However, increase in this measurement is associated with a reduction in aortoseptal angle.^7^ Inclusion of patients with more prominent basal septal hypertrophy may therefore be expected to increase sensitivity and specificity of aortoseptal angulation for the diagnosis of provocable LVOTO.

## Conclusion

Measurement of aortoseptal angulation using transthoracic echocardiography in patients with HCM is easy, reproducible, comparable to MRI, and can be calculated using standard echocardiographic software. Patients with HCM have smaller aortoseptal angles than those found in controls, where they are associated with higher peak LVOT gradient. A reduced aortoseptal angle is highly specific for provocable LVOTO and should prompt further evaluation in symptomatic patients without resting obstruction.
